# Calibration Techniques for Accurate Measurements by Underwater Camera Systems

**DOI:** 10.3390/s151229831

**Published:** 2015-12-07

**Authors:** Mark Shortis

**Affiliations:** School of Mathematical and Geospatial Sciences, RMIT University, GPO Box 2476, Melbourne 3001, Australia; mark.shortis@rmit.edu.au; Tel.: +61-399-259-628

**Keywords:** camera, calibration, underwater, refraction, accuracy, validation, stability

## Abstract

Calibration of a camera system is essential to ensure that image measurements result in accurate estimates of locations and dimensions within the object space. In the underwater environment, the calibration must implicitly or explicitly model and compensate for the refractive effects of waterproof housings and the water medium. This paper reviews the different approaches to the calibration of underwater camera systems in theoretical and practical terms. The accuracy, reliability, validation and stability of underwater camera system calibration are also discussed. Samples of results from published reports are provided to demonstrate the range of possible accuracies for the measurements produced by underwater camera systems.

## 1. Introduction

A recent report by the World Wildlife Fund [1] notes a sharp decline in marine biodiversity, caused by overfishing, coastal development and climate change. This decline is having a significant impact on the health of the marine ecosystems and threatens the survival of common seafood choices such as tuna, shrimp, whiting and salmon. The highest impact has been on these and many other highly utilised species caught in commercial or subsistence fisheries, with populations falling by 50% during 1970 to 2010.

The sustainability of wild fish stocks has been an ongoing concern that has been subject to many studies and reviews over the last few decades (for example, see [2]). Fishing has been shown to result in substantial changes in species composition and population distributions of target and non-target fish [3]. Over-fishing, especially of top level predators such as tuna and sharks, can result in unpredictable changes in marine ecosystems. In an era of increasing catch effort to maintain the dietary contribution of seafood, early detection of the impacts of over-fishing or detrimental changes in the environment is critical.

In response to declining wild fish stocks and increasing catch effort to land the same biomass, many countries have developed aquaculture industries to maintain levels of seafood dietary contribution [4]. Species such as tuna, tilapia and salmon are most commonly farmed due to their market acceptance, rapid growth and favourable food conversion rates [5]. For species subject to catch quotas, such as Southern Bluefin Tuna, the annual biomass of the catch must be estimated [6]. Once the fish are established in the aquaculture facility, monitoring of the biomass is essential for farm managers to optimise feed regimes and harvest strategies.

The age and biomass of fish can be reliably estimated based on length measurement and a length-weight or length-age regression [7,8]. When combined with spatial or temporal sampling in marine ecosystems, or counts of fish in an aquaculture cage or a trawl net, the distribution of lengths can be used to estimate distributions of or changes in biomass, and shifts in or impacts on population distributions. Underwater camera and video systems are now widely employed as a non-contact, non-invasive technique to capture accurate length information [9] and thereby estimate biomass or population distributions. Underwater camera and video systems have the further advantages that the measurements are repeatable and impartial [10], sample areas can be very accurately estimated [11] and the accuracy of the length measurements vastly improves the statistical power of the population estimates when sample counts are very low [12].

Underwater stereo-video systems have been used in the assessment of wild fish stocks with a variety of cameras and modes of operation [13,14,15,16], in pilot studies to monitor length frequencies of fish in aquaculture cages [6,17,18] and in fish nets during capture [19]. Commercial systems such as the AKVAsmart, formerly VICASS [20], and the AQ1 AM100 [18] are widely used in aquaculture and fisheries.

Marine conservation and fisheries stock assessment dominate the application of accurate measurement by underwater stereo systems, based on citations [9,14]. However there are many other applications of single camera and stereo systems reported in the literature. Stereo camera systems were used to conduct the first accurate sea bed mapping applications [21,22] and surveys of shipwrecks using either a frame [23] or towed body systems [24]. Single and stereo cameras have been used for monitoring of submarine structures, most notably to support energy exploration and extraction in the North Sea [25,26], underwater inspection of ship hulls [27] and structures [28], archaeological mapping of shipwrecks from submersibles [29], virtual modeling of archaeological sites [30], mapping of seabed topography [22,31], reconstruction of complex 3D structures [32] and inshore sea floor mapping [33,34].

A video camera has been used to measure the shape of fish pens [35] and a stereo camera has been used to map cave profiles [36]. Digital still cameras have been used underwater for mapping of artefacts in a ship wreck [37] and the estimation of sponge volumes [38]. Sea floor monitoring has also been carried out in deep water using continuously recorded stereo video cameras combined with a high resolution digital still camera [39]. A network of digital still camera images has been used to accurately characterise the shape of a semi-submerged ship hull [40].

The common factor for all of these applications of underwater imagery is a designed or specified level of accuracy. Video surveys for biomass or population distributions are directly dependent on the accuracy of the length measurements. Any inaccuracy will lead to significant errors in the estimated biomass [41] or a bias in the population distribution [12]. Other applications such as structural monitoring or seabed mapping must achieve a certain level of accuracy for the surface shape.

Calibration of any camera system is essential to achieve accurate and reliable measurements. Small errors in the perspective projection must be modelled and eliminated to prevent the introduction of systematic errors into the measurements. In the underwater environment, the calibration of the cameras is of even greater importance because the effects of refraction through the air, housing and water interfaces must be incorporated.

Compared to in-air calibration, camera calibration under water is subject to the additional uncertainty caused by attenuation of light through the housing port and water media, as well as the potential for small errors in the refracted light path due to modelling assumptions or non-uniformities in the media. Accordingly, the precision and accuracy of calibration underwater is always expected to be degraded relative to an equivalent calibration in air. Experience demonstrates that, because of these effects, underwater calibration is more likely to result in scale errors in the measurements.

## 2. Calibration Approaches 

In a limited range of circumstances calibration may not be necessary. If a high level of accuracy is not required, and the object to be measured approximates a two dimensional planar surface, a very straightforward solution is possible.

Correction lenses or dome ports such as those described in [31,42] can be used to provide a near-perfect central projection under water by eliminating the refraction effects. Any remaining, small errors or imperfections can either be corrected using a grid or graticule placed in the field of view, or simply accepted as a small deterioration in accuracy. The correction lens or dome port has the further advantage that there is little, if any, degradation of image quality near the edges of the port. Plane camera ports exhibit loss of contrast and intensity at the extremes of the field of view due to acute angles of incidence and greater apparent thickness of the port material.

This simplified approach has been used, either with correction lenses or a pre-calibration of the camera system, to carry out two dimensional mapping. A portable control frame with a fixed grid or target reference is imaged before deployment or placed against the object to measured, to provide both calibration corrections as well as position and orient the camera system relative to the object. Typical applications of this approach are ship wreck mapping [23], sea floor characterisation surveys [31], length measurements in aquaculture [17] and monitoring of sea floor habitats [43].

However if accuracy is a priority, and especially if the object to be measured is a three dimensional surface, then a comprehensive calibration is essential. The correction lens approach assumes that the camera is a perfect central projection and that the entrance pupil of the camera lens coincides exactly with the centre of curvature of the correction lens. Any simple correction approach, such as a graticule or control frame placed in the field of view, will be applicable only at the same distance. Any significant extrapolation outside of the plane of the control frame will inevitably introduce systematic errors.

The alternative approach of a comprehensive calibration translates a reliable technique from in air into the underwater environment. Close range calibration of cameras is a well-established technique that was pioneered by [44], extended to include self-calibration of the camera(s) by [45] and subsequently adapted to the underwater environment [46,47]. The mathematical basis of the technique is described in [48].

**Figure 1:**
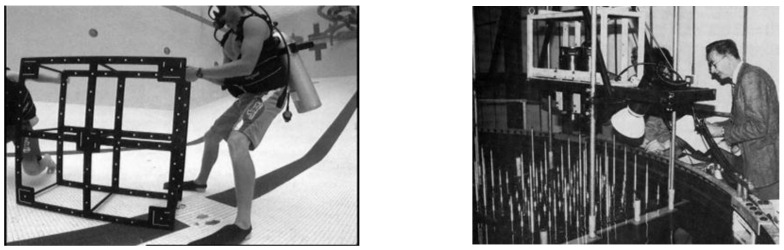
Typical portable calibration fixture ((**Left**), courtesy of NOAA) and test range ((**Right**), from [25]).

**Figure 2:**
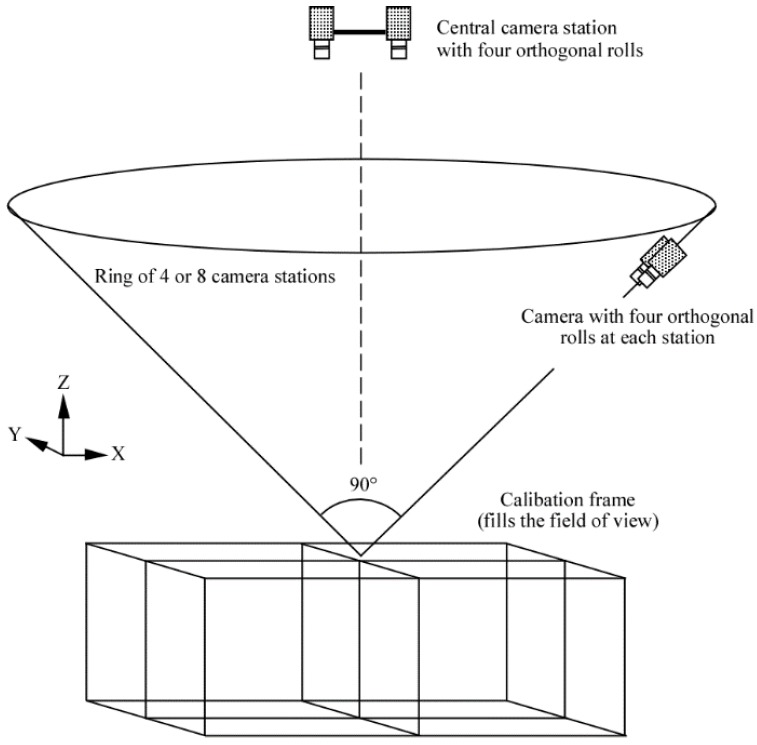
The ideal geometry for a self-calibration network.

**Figure 3:**
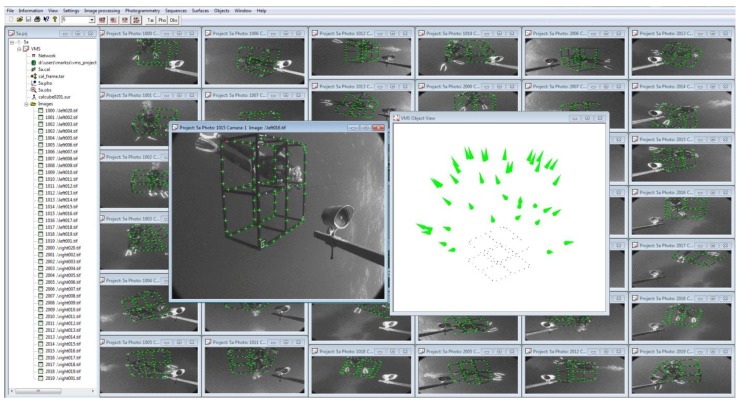
A full set of calibration images from an underwater stereo-video system, processed using Vision Measurement System (www.geomsoft.com/VMS). Both the cameras and the object have been rotated to acquire the convergent geometry of the network.

The essence of this approach is to capture multiple, convergent images of a fixed calibration range or portable calibration fixture (see Figure 1) to determine the physical parameters of the camera calibration. A typical calibration range or fixture is based on discrete targets to precisely identify measurement locations throughout the camera fields of view from the many photographs (see Figure 1). The targets may be circular dots or the corners of a checkerboard. Coded targets or checkerboard corners on the fixture can be automatically recognised using image analysis techniques [49,50] to substantially improve the efficiency of the measurements and network processing. The ideal geometry and a full set of images for a calibration fixture are shown in Figure 2 and Figure 3 respectively.

A fixed test range, such as the “Manhattan” object shown in Figure 1, has the advantage that accurately known target coordinates can be used in a pre-calibration approach, but the disadvantage that the camera system has to be transported to the range and then back to the deployment location. In comparison, accurate information for the positions of the targets on a portable calibration fixture is not required, as coordinates of the targets can be derived as part of a self-calibration approach. Hence it is immaterial if the portable fixture distorts or is dis-assembled between calibrations, although the fixture must retain its dimensional integrity during the image capture. Scale within the 3D measurement space is determined by introducing distances measured between pre-identified targets into the self-calibration network [51]. The known distances between the targets must be reliable and accurate, so known lengths are specified between targets on the rigid arms of the fixture or between the corners of the checkerboard.

In practice, cameras are most often pre-calibrated using a self-calibration network and a portable calibration fixture in a venue convenient to the deployment. The refractive index of water is insensitive to temperature, pressure or salinity [31], so the conditions prevailing for the pre-calibration can be assumed to be valid for the actual deployment of the system to capture measurements. The assumption is also made that the camera configurations, such as focus and zoom, and the relative orientation for a multi camera system, are locked down and undisturbed. A close proximity between the locations of the calibration and the deployment minimises the risk of a physical change to the camera system.

The process of self-calibration of underwater cameras is straightforward and rapid. The calibration can take place in a swimming pool, in an on-board tank on the vessel or, conditions permitting, adjacent to, or beneath, the vessel. The calibration fixture can be held in place and the cameras maneuvered around it, or the calibration fixture can be manipulated whilst the cameras are held in position, or a combination of both approaches can be used (see Figure 3). For example, a small 2D checkerboard may be manipulated in front of an ROV stereo-camera system held in a tank. A large, towed body system may be suspended in the water next to a wharf and a large 3D calibration fixture manipulated in front of the stereo video cameras. In the case of a diver-controlled stereo-camera system, a 3D calibration fixture may be tethered underneath the vessel and the cameras moved around it.

There are very few examples of *in-situ*, self-calibrations of camera systems, because this type of approach is not readily adapted to the dynamic and uncontrolled underwater environment. Nevertheless, there are some examples of a single camera or stereo-pair *in-situ* self-calibration [27,35,37,38]. In most cases a pre-calibration is conducted to determine an initial estimate of the calibration of the camera system.

## 3. Calibration Algorithms

**Figure 4:**
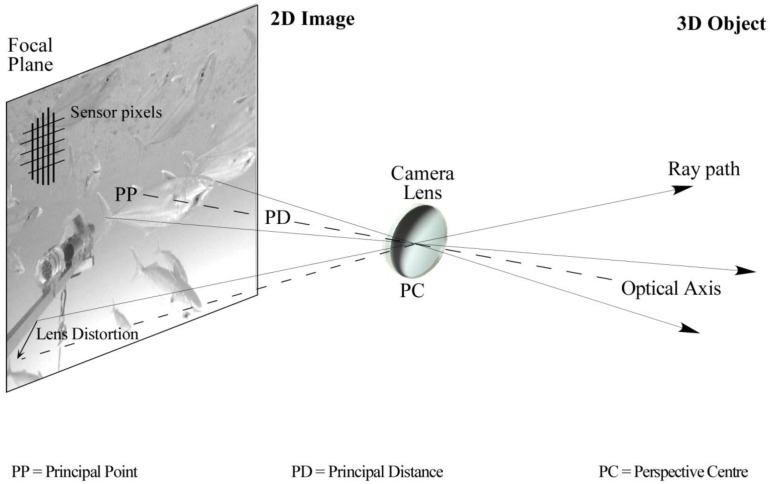
The geometry of perspective projection based on physical calibration parameters.

Calibration of a camera system is necessary for two reasons. First, the internal geometric characteristics of the cameras must be determined [44]. In photogrammetric practice, camera calibration is most often defined by physical parameter set (see Figure 4) comprising principal distance, principal point location, radial [52] and decentring [53] lens distortions, plus affinity and orthogonality terms to compensate for minor optical effects [54,55]. The principal distance is formally defined as the separation, along the camera optical axis, between the lens perspective centre and the image plane. The principal point is the intersection of the camera optical axis with the image plane.

**Figure 5:**
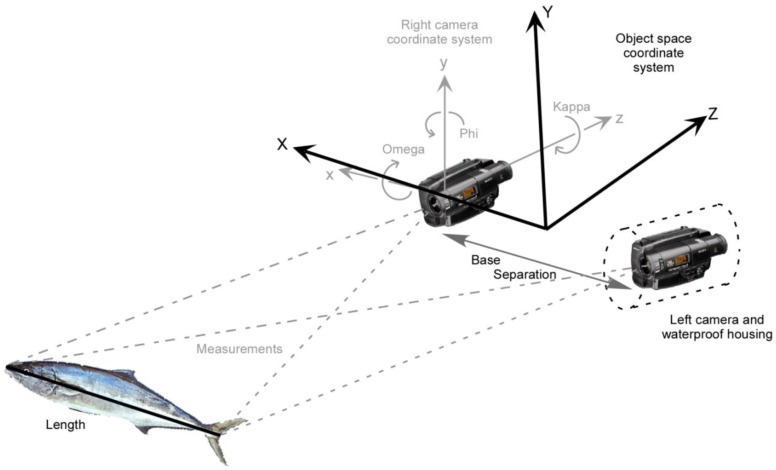
Schematic view of a stereo-image measurement of a length from 3D coordinates.

Second, the relative orientation of the cameras with respect to one another, or the exterior orientation with respect to an external reference, must be determined. Also known as pose estimation, both the location and orientation of the camera(s) must be determined. For the commonly used approach of stereo cameras, the relative orientation effectively defines the separation of the perspective centres of the two lenses, the pointing angles (omega and phi rotations) of the two optical axes of the cameras and the roll angles (kappa rotations) of the two focal plane sensors (see Figure 5).

**Figure 6:**
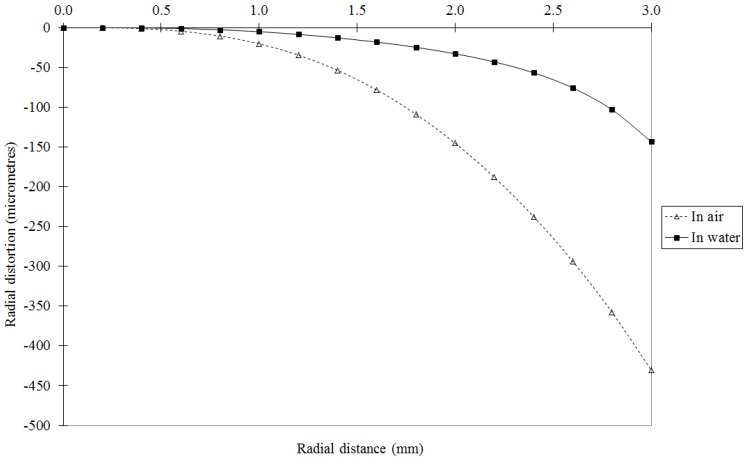
Comparison of radial lens distortion from in-air and in-water calibrations of a GoPro Hero4 camera operated in HD video mode.

**Figure 7:**
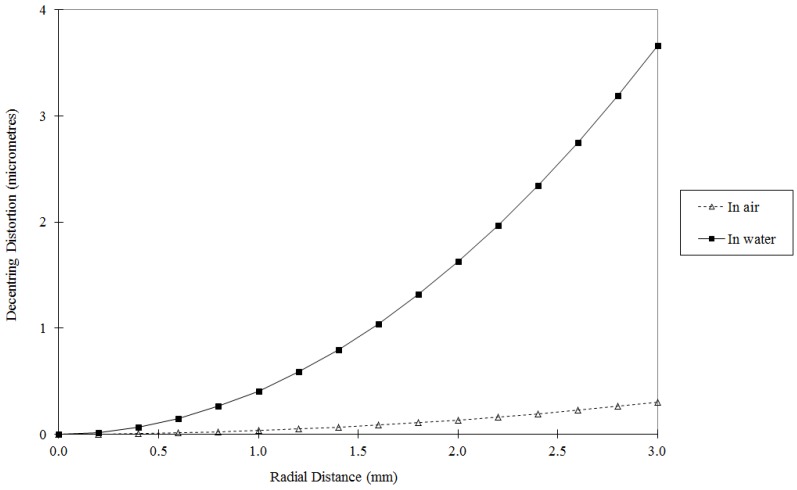
Comparison of decentring lens distortion from in-air and in-water calibrations of a GoPro Hero4 camera operated in HD video mode. Note the much smaller range of distortion values (vertical axis) compared to Figure 6.

In the underwater environment the effects of refraction must be corrected or modelled to obtain an accurate calibration. The entire light path, including the camera lens, housing port and water medium, must be considered. By far the most common approach is to correct the refraction effects using absorption by the physical camera calibration parameters. Assuming that the camera optical axis is approximately perpendicular to a plane or dome camera port, the primary effect of refraction through the air-port and port-water interfaces will be radially symmetric around the principal point [56]. This primary effect can be absorbed by the radial lens distortion component of the calibration parameters. Figure 6 shows a comparison of radial lens distortion from calibrations in air and in water for the same camera. There will also be some small, asymmetric effects caused by, for example, alignment errors between the optical axis and the housing port, and perhaps non-uniformities in the thickness or material of the housing. These secondary effects can be absorbed by calibration parameters such as the decentring lens distortion and the affinity term. Figure 7 shows a comparison of decentring lens distortion from calibrations in air and in water of the same camera. Similar changes in the lens distortion profiles are demonstrated in [46,57].

Table 1 shows some of the calibration parameters for the in air and in water calibrations of two GoPro Hero4 camera. The ratios of the magnitudes of the parameters indicate whether there is a contribution to the refractive effects. As could be expected, for a plane housing port the principal distance is affected directly, whilst changes in parameters such as the principal point location and the affinity term may include the combined influences of secondary effects, correlations with other parameters and statistical fluctuation. These results are consistent for the two cameras, consistent with other cameras tested, and [57,58] present similar outcomes from in air *versus* in water calibrations for flat ports. Very small percentage changes to all parameters, including the principal distance, are reported in [59] for housings with dome ports. Increases in principal distance of 1% to 25% for dome and flat ports are reported in [32]. All of these results are generally in accord with the expected physical model of the refraction.

**Table 1:** Comparison of parameters from in air and in water calibrations for two GoPro Hero4 camera used in HD video mode.

Camera	GoPro Hero4 #1			GoPro Hero4 #2		
Parameter	In Air	In Water	Ratio	In Air	In Water	Ratio
PPx (mm)	0.080	0.071	0.88	−0.032	−0.059	1.82
PPy (mm)	−0.066	−0.085	1.27	−0.143	−0.171	1.20
PD (mm)	3.676	4.922	1.34	3.658	4.898	1.34
Affinity	−6.74E−03	−6.71E−03	1.00	−6.74E−03	−6.84E−03	1.01

The disadvantage of the absorption approach for the refractive effects is that there will always be some systematic errors which are not incorporated into the model. The effect of refraction invalidates the assumption of a single projection centre for the camera [60], which is the basis for the physical parameter model. The errors are most often manifest as scale changes when measurements are taken outside of the range used for the calibration process. Experience over many years of operation demonstrates that, if the ranges for the calibration and the measurements are commensurate, then the level of systematic error is generally less than the precision with which measurements can be extracted. This masking effect is partly due to the elevated level of noise in the measurements, caused by the attenuation and loss of contrast in the water medium.

The alternative to the simple approach of absorption is the more complex process of geometric correction, effectively an application of ray tracing of the light paths through the refractive interfaces. A two phase approach is developed in [61] for a stereo camera housing with concave lens covers. An in air calibration is carried out first, followed by an in water calibration that introduces 11 lens cover parameters such as the centre of curvature of the concave lens and, if not known from external measurements, refractive indices for the lens covers and water. A more general geometric correction solution is developed for plane port housings in [62]. Additional unknowns in the solution are the distance between the camera perspective centre and the housing, and the normal of the plane housing port, whilst the port thickness and refractive indices must be known. Using ray tracing, [63] develops a general solution to refractive surfaces that, in theory, can accommodate any shape of camera housing port. The shape of the refractive surface and the refractive indices must be known.

A variation on the geometric correction is the perspective centre shift or virtual projection centre approach. A specific solution for a planar housing port is developed in [64]. The parameters include the standard physical parameters, the refractive indices of glass and water, the distance between the perspective centre and the port, the tilt and direction of the optical axis with respect to the normal to the port, and the housing interface thickness. A modified approach neglects the direction of the optical axis and the thickness of thin ports, as these factors can be readily absorbed by the standard physical parameters. Again a two phase process is required, first a “dry” calibration in air and then a “wet” calibration in water [64]. A similar principle is used in [65], also with a two phase calibration approach.

The advantage of these techniques is that, without the approximations in the models, the correction of the refractive effects is exact. The disadvantages are the requirements for two phase calibrations and known data such as refractive indices. Further, in some cases the theoretical solution is specific to a housing type, whereas the absorption approach has the distinct advantage that it can be used with any type of underwater housing.

As well as the common approaches described above, some other investigations are worthy of note. The Direct Linear Transformation (DLT) algorithm [66] is used with three different techniques in [67]. The first is essentially an absorption approach, but used in conjunction with a sectioning of the object space to minimise the remaining errors in the solution. A double plane correction grid was applied in the second approach. In the last technique a formal refraction correction model is included with the requirements that the camera-to-interface distance and the refractive index must be known. The solutions presented in [67] suggest that both the absorption and refraction correction approaches can be used successfully in association with different calibration algorithms, either linear models such as DLT [66], multi-stage linear solutions [68,69] or non-linear models based on the standard physical parameters [44].

A review of refraction correction methods for underwater imaging is given in [60]. The perspective camera model, ray-based models and physical models are analysed, including an error analysis based on synthetic data. The analysis demonstrates that perspective camera models incur increasing errors with increasing distance and tilt of the refractive surfaces, and only the physical model of refraction correction permits a complete theoretical compensation.

Once the camera calibration is established, single camera systems can be used to acquire measurements when used in conjunction with reference frames [29] or sea floor reference marks [37]. For multi-camera systems the relative orientation is required as well as the camera calibration. The relative orientation can be included in the self-calibration solution as a constraint [70] or can be computed as a post-process based on the camera positions and orientations for each set of synchronised exposures [47]. In either case, it is important to detect and eliminate outliers, usually caused by lack of synchronisation, that would otherwise unduly influence the calibration solution or the relative orientation computation. Outliers caused by synchronisation effects are more common for systems based on camcorders or video cameras in separate housings, which typically use an external device such as a flashing LED light to synchronise the images to within one frame [47].

In the case of post-processing, the exterior orientations for the sets of synchronised exposures are initially in the frame of reference of the calibration fixture, so each set must be transformed into a local frame of reference with respect to a specific baseline between the cameras. In the case of stereo cameras, the local frame of reference is adopted as the centre of the baseline between the camera perspective centres, with the axes aligned with the baseline direction and the mean optical axis pointing direction (see Figure 5). The final parameters for the precise relative orientation are adopted as the mean values for all sets in the calibration network, after any outliers have been detected and eliminated.

## 4. Calibration Reliability and Stability

The reliability and accuracy of the calibration of underwater camera systems is dependent on a number of factors. Chief amongst the factors are the geometry and redundancy for the calibration network. A high level of redundant information, provided by many target image observations on many exposures, produces high reliability so that outliers in the image observations can be detected and eliminated. An optimum three dimensional geometry is essential to minimise correlations between the parameters and ensure that the camera calibration is an accurate representation of the physical model [45]. However it should be noted that it is not possible to eliminate all correlations between the calibration parameters. Correlations are always present between the three radial distortion terms and between the principal point and two decentring terms.

The accuracy of the calibration parameters is enhanced if the network of camera and target locations meets the following criteria:

(1) The camera and target arrays are three dimensional in nature. Two dimensional arrays are a source of weak network geometry. Three dimensional arrays minimise correlations between the internal camera calibration parameters and the external camera location and orientation parameters.
(2) The many, convergent camera views approach a 90° intersection at the centre of the target array. A narrowly grouped array of camera views will produce shallow intersections, weakening the network and thereby decreasing the confidence with which the calibration parameters are determined.
(3) The calibration fixture or range fills the field of view of the camera(s) to ensure that image measurements are captured across the entire format. If the fixture or range is small and centred in the field of view then the radial and decentring lens distortion profiles will be defined very poorly because measurements are captured only where the signal is small in magnitude.
(4) The camera(s) are rolled around the optical axis for different exposures so that 0°, 90°, 180° and 270° orthogonal rotations are spread throughout the calibration network. A variety of camera rolls in the network also minimises correlations between the internal camera calibration parameters and the external camera location and orientation parameters.

If these four conditions are met, the self-calibration approach can be used to simultaneously and confidently determine the camera calibration parameters, camera exposure locations and orientations, and updated target coordinates [45].

In recent years there has been an increasing adoption of a calibration technique using a small 2D checkerboard and a freely available Matlab solution [71]. The main advantages of this approach are the simplicity of the calibration fixture and the rapid measurement and processing of the captured images, made possible by the automatic recognition of the checkerboard pattern [50]. A practical guide to the use of this technique is provided in [72].

However the small size and 2D nature of the checkerboard limits the reliability and accuracy of measurements made using this technique [41]. The technique is equivalent to a test range calibration rather than a self-calibration, because the coordinates of the checkerboard corners are not updated. Any inaccuracy in the coordinates, especially if the checkerboard has variations from a true 2D plane, will introduce systematic errors into the calibration. Nevertheless, the 2D fixture can produce a calibration suitable for measurements at short ranges and with modest accuracy requirements. AUV and diver operated stereo camera systems pre-calibrated with this technique have been used to capture fish length measurements [16,72] and tested for the 3D re-construction of artefacts [59].

The stability of the calibration for underwater camera systems has been well documented in published reports [73,74]. As noted previously, the basic camera settings such as focus and zoom must be consistent between the calibration and deployments, usually ensured through the use of tape or a locking screw to prevent the settings from being inadvertently altered. For cameras used in air, other factors are handling of the camera, especially when the camera is rolled about the optical axis or a zoom lens is being employed, and the quality of the lens mount. Any distortion of the camera body or movement of the lens or optical elements will result in variation of the relationship between the perspective centre and the imager at the focal plane, which will disturb the calibration [75]. Fixed focal length lenses are preferred over zoom lenses to minimise the instabilities.

However the most significant sensitivity for the calibration stability of underwater camera systems is the relationship between the camera lens and the housing port. Rigid mountings of the cameras in the housings is critical to ensure that the total optical path from the image sensor to the water medium is consistent [73]. Testing and validation has shown that the camera calibration is only reliable if the cameras in the housings are mounted on a rigid connection to the camera port [74]. This applies to both within a single deployment and between multiple, separate deployments of the camera system. Unlike correction lenses and dome ports, a specific position and alignment within the housing is not necessary, but the distance and orientation of the camera lens relative to the housing port must be consistent. The most reliable option is a direct, mechanical linkage between the camera lens and the housing port that can consistently re-create the physical relationship. The consistency of distance and orientation is especially important for portable camcorders because they must be regularly removed from the housings to retrieve storage media and replenish batteries.

**Figure 8:**
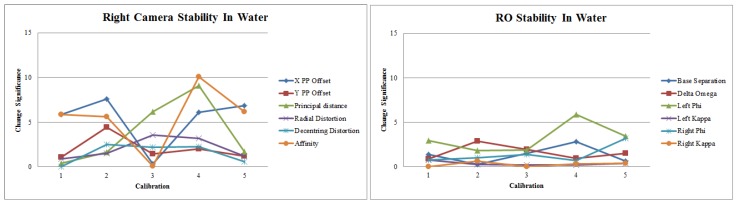
Stability of the right camera calibration parameters (**Left**) and the relative orientation parameters (**Right**) for a GoPro Hero 2 stereo-video system. The vertical axis is the change significance of individual parameters between consecutive calibrations [73].

Finally, for multi-camera systems, in air or in water, the camera housings must have a rigid mechanical connection to a base bar to ensure that the separation and relative orientation of the cameras is also consistent. Perturbation of the separation or relative orientation often results in apparent scale errors which can be readily confused with refractive effects. Figure 8 shows some results of repeated calibrations of a GoPro Hero 2 stereo-video system. The variation in the parameters between consecutive calibrations demonstrates a comparatively stable relative orientation but a more unstable camera calibration caused by a non-rigid mounting of the camera in the housing. Note that these tests were based on video frames captured with a motionless camera and calibration object in order to avoid any motion effects from the rolling shutter used by GoPro cameras [76]. Rapid motion should be avoided for GoPro cameras when capturing video for calibration or measurement.

## 5. Calibration and Validation Results

The first evaluation of a calibration is generally the internal consistency of the network solution that is used to compute the calibration parameters, camera locations and orientations, and if applicable, updated target coordinates. The “internal” indicator is the Root Mean Square (RMS) error of image measurement, a metric for the internal “fit” of the least squares estimation solution [48]. Note that in general the measurements are based on an intensity weighted centroid to locate the centre of each circular target in the image [77].

To allow comparison of different cameras with different spacing of the light sensitive elements in the CMOS or CCD imager, the RMS error is expressed in fractions of a pixel. In ideal conditions in air, the RMS image error is typically in the range of 0.03–0.1 pixels [77]. In the underwater environment, the attenuation of light and loss of contrast, along with small non-uniformities in the media, degrades the RMS error into the range of 0.1–0.3 pixels (see Table 2). This degradation is a combination of a larger statistical signature for the image measurements and the influence of small, uncompensated systematic errors. In conditions of poor lighting or poor visibility the RMS error deteriorates rapidly [72].

The second metric that is commonly used to compare the calibration, especially for in air operations, is the proportional error, expressed as the ratio of the magnitude of the average precision of the 3D coordinates of the targets to the largest 3D Euclidian distance contained within the volume of the object. This “external” indicator provides a standardised, relative measure of precision in the object space. In the circumstance of a camera calibration, the largest 3D distance is the diagonal span of the test range volume, or the diagonal span of the volume envelope of all imaged locations of the calibration fixture. Whilst the RMS image error may be favourable, the proportional error may be relatively poor if the object is contained within a small volume or the geometry of the calibration network is poor. Table 2 presents a sample of some results for the precision of calibrations. It is evident that the proportional error can vary substantially, however an average figure is approximately 1:5000.

**Figure 9:**
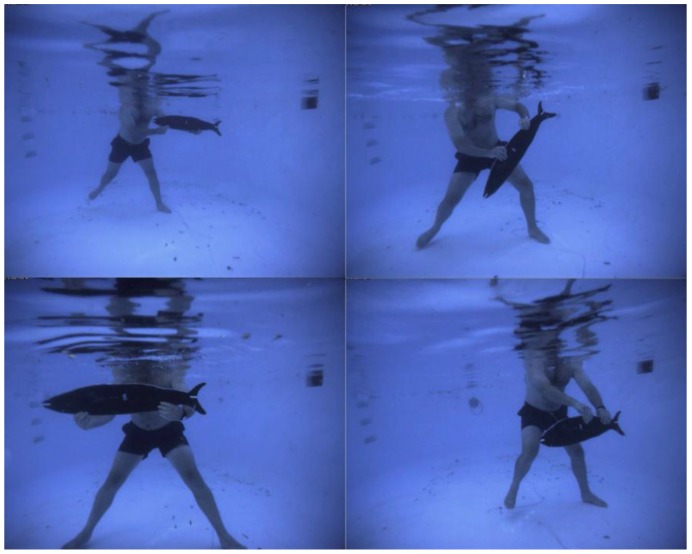
Example of a fish silhouette validation in a swimming pool (courtesy of Prof. E. S. Harvey).

As a consequence of the potential misrepresentation by proportional error, independent testing of the accuracy of underwater camera systems is essential to ensure the validity of 3D locations, length, area or volume measurements. For stereo and multi camera systems, the primary interest is length measurements that are subsequently used to estimate biomass or age. One validation technique is to use known distances on the rigid components of the calibration fixture [6], however this has some limitations. As already noted, the circular, discrete targets are dissimilar to the natural feature points of a fish snout or tail, and are measured by different techniques. The variation in size and angle of the distance on the calibration fixture may not correlate well with the size and orientation of fish when measured. In particular, measurements of fish are often taken at greater ranges than that of the calibration fixture, partly due to expediency in surveys and partly because the calibration fixture must be close enough to the cameras to fill a reasonable portion of the field of view. Given the approximations in the refraction models, it is important that accuracy validations are carried out at ranges greater than the average range to the calibration fixture. Further, it has been demonstrated that the accuracy of length measurements is dependent on the separation of the cameras in a multi camera system [41] and significantly affected by the orientation of the fish relative to the cameras [47,78]. Accordingly, validation of underwater video measurement systems is typically carried out by introducing a known length, either a rod or a fish silhouette, which is measured manually at a variety of ranges and orientations within the field of view (see Figure 9).

**Table 2:** A sample of some published results for the precision of underwater camera calibrations. Note that [35] used observations of a mobile fish pen and the measurements used by [61] were made to the nearest whole pixel.

Technique	RMS Image Error (pixels)	RMS XYZ Error (mm)	Proportional Error
Absorption [47,73]	0.1–0.3	0.1–0.5	1:3000–1:15,000
Absorption [35]	0.3	40–200	1:500
Geometric correction [61]	1.0	10	1:210
Perspective shift [64]	0.3	2.0	1:1000
Absorption [40]	0.2–0.25	1.9	1:32,000

In the best case scenario of clear visibility and high contrast targets, the RMS error of validation measurements is typically less than 1 mm over a length of 1 m, equivalent to a length accuracy of 0.1%. In realistic, operational conditions using fish silhouettes or validated measurements of live fish, length measurements have an accuracy of 0.2% to 0.7% [6,11,41,64,78]. The accuracy is somewhat degraded if a simple correction grid is used [17] or a simplified calibration approach is adopted [72]. A sample of published validation results is given in Table 3.

**Table 3:** A sample of some published results for the validation of underwater camera calibrations.

Technique	Validation	Percentage Error
Absorption [47]	Length measurement of silhouettes or rods throughout the volume	0.2%–0.7%
Lens distortion grid [17]	Caliper measurements of Chinook Salmon	1.5%
Absorption [6]	Caliper measurements of Southern Bluefin Tuna	0.2%
Perspective shift [64]	Flat reference plate and straight line re-construction	0.4%
Absorption [40]	Similarity transformation between above and below water networks	0.3%
Radial lens distortion correction [72]	Distances on checkerboard	0.9%–1.5%
Absorption [41]	Length measurements of a rod throughout the volume	0.5%
Perspective shift [65]	Flat reference plate and distance between spheres	0.4%–0.7%

Validations of biomass estimates of Southern Bluefin Tuna measured in aquaculture pens and sponges measured in the field have shown that volume or biomass can be estimated with an accuracy of the order of a few percent. The Southern Bluefin Tuna validation was based on distances such as body length and span, made by a stereo-video system and compared to a length board and caliper system of manual measurement. Each Southern Bluefin Tuna in a sample of 40 fish was also individually weighed. The stereo-video system produced an estimate of better than 1% for the total biomass [6]. Triangulation meshes on the surface of simulated and live specimens were used to estimate the volume of sponges. The resulting errors were 3%–5%, and no worse than 10%, for individual sponges [38]. Greater variability is to be expected for the estimates of the sponge volumes, because of the uncertainty associated with the assumed shape of the unseen substrate surface beneath each sponge.

By the very nature of conversion from length to weight, errors can be amplified significantly. Typical regression functions are power series with a near cubic term [7,8,41]. Accordingly, inaccuracies in the calibration and the precision of the measurement may combine to produce unacceptable results. A simulation is employed by [41] to demonstrate clearly that the predicted error in the biomass of a fish, based on the error in the length, deteriorates rapidly with range from the cameras, especially with a small 2D calibration fixture and a narrow separation between the stereo cameras. Errors in the weight in excess of 10% are possible, reinforcing the need for validation testing throughout the expected range of measurements. Validation at the most distant ranges, where errors in biomass can approach 40%, is critical to ensure that an acceptable level of accuracy is maintained.

## 6. Conclusions

This paper has presented a review of different calibration techniques that incorporate the effects of refraction from the camera housing and the water medium. Calibration of underwater camera systems is essential to ensure the accuracy and reliability of measurements of marine fauna, flora or artefacts. Calibration is a key process to ensure that the analysis of biomass, population distribution or dimensions is free of systematic errors.

Irrespective of whether an implicit absorption or an explicit refractive model is used in the calibration of underwater camera systems, it is clear from the sample of validation results that an accuracy of the order of 0.5% of the measured dimensions can be achieved. Less favourable results are likely when approximate methods, such as 2D planar correction grids, are used. The configuration of the underwater camera system is a significant factor that has a primary influence on the accuracy achieved. However the advantage of photogrammetric systems is that the configuration can be readily adapted to suit the required measurement accuracy.

Further investigation of different calibration algorithms is warranted to assess the merits of the various approaches. Otherwise confounding factors, such as the size of the calibration fixture, the range of locations and the image measurement technique, should be common to all calibration techniques to gain a valid comparison. The evaluation of such testing should be based on a consistent and rigorous validation process to ensure that all techniques are compared on the same basis.
